# In Vitro Comparative Quality Assessment of Different Brands of Doxycycline Hyclate Finished Dosage Forms: Capsule and Tablet in Jimma Town, South-West Ethiopia

**DOI:** 10.1155/2021/6645876

**Published:** 2021-02-09

**Authors:** Woldemichael Abraham, Habtamu Abuye, Selass Kebede, Sultan Suleman

**Affiliations:** ^1^Department of Pharmacy, College of Medicine and Health Sciences, Wachemo University, Hosaena, Ethiopia; ^2^Jimma University Laboratory of Drug Quality, Department of Pharmaceutical Quality Analysis and Regulatory Affairs, School of Pharmacy, College of Health Sciences, Jimma University, Jimma, Ethiopia

## Abstract

**Background:**

Persistent postmarket quality evaluation helps produce clear information on the current quality status of the different brands of a given drug and hence introduces a biopharmaceutical and therapeutically equivalent list of the products to the prescribers and users of it. This in turn facilitates access to essential medicines by breaking the high-cost barrier imposed by a few expensive brands of the product. This study was aimed at determining the quality and evaluating the equivalence of doxycycline hyclate capsules and tablets in Jimma, Ethiopia.

**Methods:**

Ten brands of doxycycline hyclate capsules and tablets were tested for product identity, dosage uniformity, assay, and *in vitro* dissolution; and tablets were tested for friability and hardness.

**Results:**

All investigated brands of doxycycline complied with the USP for dosage uniformity, an assay of the active ingredient, and single-point dissolution tests. One brand, D09, failed both hardness and friability tests. Comparisons of dissolution profiles applying fit factors confirmed that only brands D04, D06, and D07 had similarities with the innovator. Ratio test approaches also showed that significant variability exists between test products and comparators. Weibull model was found to provide the best adjustment curve for all brands, from model-dependent approaches employed for explaining the overall release of drug from the dosage forms.

**Conclusions:**

Doxycycline is a biowaiver product. Hence, *in vitro* dissolution evaluation suffices its market approval. In this quality assessment study, however, the samples passed quality control tests, except D09 brand which failed friability; it has been revealed that five out of eight brands had problems with interchangeability. Only three doxycycline hyclate brands were found to be equivalent to the comparators.

## 1. Introduction

The choice of drugs in the management of any disease is an important aspect being considered by healthcare practitioners, patients, government, and health insurance companies [1, 2]. As cost is one of the barriers to essential medicines, especially in low-income countries that have to pay out of pocket for medicines, the use of generic medicines solves the problem in part because generic medicines are 20–90% cheaper than their counterpart innovator medicines [3]. Since the use of generic medicines provides substantial savings to healthcare systems in recent years, many governments and third-party payers have advocated the utilization of generic medicines as a means of confronting the escalation of healthcare expenditure in general and medicine expenditure in particular, by instigating various policies, initiatives, and strategies [4].

The concern about lowering health care costs has resulted in a tremendous increase in the use of generic drug products. However, providing generic drugs from multiple sources into the healthcare system as a means of reducing healthcare costs is associated with substandard, spurious, falsely labeled, falsified, and counterfeit medicines [5].

Currently, it is estimated that 10–15% of the global drugs supplied are counterfeit. The prevalence is higher in developing countries, in Africa, and in parts of Asia and Latin America where up to 30–60% of drugs on the market are counterfeit [6]. Among the medicines, antibiotics account for 28% of global counterfeit medicines [6]. These problems have resulted in a weak therapeutic efficiency and development of dire resistant strains [7]. There is, therefore, a need to routinely assess the pharmaceutical quality of drugs.

On the other hand, advocating strategy for utilization of generic medicines, as seen from the pilot study, was wrongly interpreted by the clients (which is most likely sown and cultivated by drug promoters and intensified through health service givers). As the informal market assessment of Jimma Town indicated, there was a huge price difference (18.57 times) among different brands of doxycycline 100 mg capsules and tablets in licensed pharmaceutical retail outlets. Patients, especially from private health institutions, doubt the efficacy, safety, and quality of the cheaper products. They were convinced to buy the prescribed brand even though he/she has an economical constraint to afford. When they were counseled to take an alternative affordable one, their feeling showed an unwillingness to adhere to that generic product. Pharmacists were seen acting as a seller only instead of discussing the drug issues that the patients have. Both side problems reside on fewer/absence of published studies that inform and assure the patients and the health professionals concerning the performance of different drug brands.

Few studies conducted in Ethiopia on comparative *in vitro* bioequivalence evaluation of different drug brands reported that 62.5% of brands of amoxicillin capsules were not interchangeable with the innovator [8], only one out of five amoxicillin capsule had a similar dissolution profile with the innovator and can be considered bioequivalent and interchangeable [9], and 10 products of co-trimoxazole tablets were investigated; the cheaper ones exhibited delayed release during dissolution testing and they released smaller amount of drug compared to the others [10].

Therefore, it is necessary to compare the existing brands of drug products in order to identify the brands that fit their purpose and can be used interchangeably with the comparator (innovator product).

This paper tried to show mainly the performances of different brands of doxycycline 100 mg capsules and tablets found in the Jimma market. Since doxycycline hyclate is a BCS-І, biowaiver product; that is, *in vitro* quality evaluation result only suffices to decide on the *in vivo* pharmacokinetic properties of the product [11].

Doxycycline is a semisynthetic second-generation tetracycline that came into use in 1967. It is better absorbed after oral administration than first-generation counterparts due to its higher lipid solubility, freely soluble in water and methanol, sparingly soluble in ethanol (96 %), and BCS class І product for which biowaiver is applied [11, 12]. The drug is chosen for its broad spectrum, high activity against nearly all Gram-positive and Gram-negative anaerobic and aerobic bacteria, mycoplasmas, *Chlamydiae*, *Rickettsiae*, and some Protozoa [13].

Doxycycline demonstrates immune-modulating activities that have been used in the treatment of numerous inflammatory conditions mediated by matrix metalloproteinase (MMP-9) and recover inflammatory biomarkers in patients with abdominal aortic aneurysms. It helps to prevent periodontitis and acute coronary syndromes. In recent times, the advancement in the study of tetracycline has developed due to their ability to inhibit matrix metalloproteinase (MMPs) in a variety of cancers such as breast, colorectal, osteosarcoma, melanoma, leukemia, and prostate cancers [14]. It has shown favorable effects in trial models of pulmonary fibrosis, emphysema, asthma, and acute lung injury [15]. It also provides antiresorption results from the inhibition of clastic cells and prevents tissue breakdown by the inhibition of mammalian collagenases. It blocks excess of tissue collagenases which is present in periodontitis, consequently leading to enhanced formation of bone and collagen [16].

Doxycycline is a widely used antibiotic among its class listed in the Essential Medicines List of Ethiopia [17]. Typhus is the most common among the indications in Ethiopia because the hygiene and sanitation conditions are conducive to the vector of the disease, lice, and increase the need for doxycycline hyclate. A study in Kaliti Prison, Addis Ababa, revealed that the serological prevalence of typhus fever was 26.3% [18]. In addition to its numerous indications, empirical treatment strategy and nonspecific laboratory test (Weil-Felix in typhus case) have increased the consumption of doxycycline even more than the real demand [19]. This may attract the attention of counterfeiters since the counterfeiting act is focused in highly prescribed or demanded products of a given country or geographical area [20].

Therefore, the present study is mainly aimed at comparison of the different brands of doxycycline 100 mg capsule and tablet with the comparators, and in the way, it tried to identify whether there were poor quality samples.

## 2. Methods and Materials

### 2.1. Materials

Doxycycline USP Standard (15008, Lot II) was kindly donated from Cadila Pharmaceuticals Ethiopia. Purified ultrapure water was obtained by water purification system (Thermo Fischer Scientific, USA, 18.2 MΩ cm at 25°C) which is found in Jimma University Laboratory of Drug Quality (JULaDQ) Jimma, Ethiopia. All other chemicals used in this study were of analytical grade and used as received.

### 2.2. Sample Collection

All available ten brands of doxycycline capsules and tablets, each with a label claim of 100 mg, were purchased from licensed drug retail outlets in Jimma Town, Ethiopia. When arriving at drug outlets, we informed the purpose and asked to buy doxycycline capsules and tablets available for sale which we did not obtain during our previous collection regardless of their price range. During sample collection, all the necessary information about the samples was recorded in a standard report form. Almost all drug retail outlets in Jimma Town were visited and only 10 brands were obtained. If a drug outlet had more than one brand, then all the different brands were taken. The experimental part of the work was undertaken at Jimma University Drug Quality Laboratory (JuLaDQ), and the study was performed before product expiration dates. Comparator product selection was based on standard guidelines [21]. Detailed information on doxycycline capsules and tablets included in the study is given in Table 1. Simple codes were given for the products.

### 2.3. Instruments

HPLC (Agilent 1260 Series, Darmstadt, Germany), Analytical Balance (Mettler Toledo, Greifensee, Switzerland), RC-6D Dissolution Apparatus (Apparatus 1 and Apparatus 2; Tian Jin Optical Instruments, Tianjin, China), UV-Vis Spectrophotometer (Cecil Instruments, Cambridge, United Kingdom), Hardness Tester (Pharma Test, Hainburg, Germany), Friability Tester (Pharma Test), and water purification system (Thermo Scientific, Model-7143, Waltham, MA, USA) Thermometer, Sonicator, Bath UltrasonicElma®, Suction Vacuum Pump (Gast Manufacturing Inc.), and standard laboratory glassware were used for the study.

### 2.4. Chemicals and Reagents

Doxycycline Reference Standard (assay: 98.99%, WS/15008, Lot II), tetrabutylammonium hydrogen sulfate, tertiary butyl alcohol, and edetate disodium were kindly gifted by Cadila Pharmaceuticals Ethiopia. Sodium hydroxide 50%, potassium phosphate monobasic (KH_2_PO_4_) 99–101%, ultrapure water, hydrochloric acid (36%, 11.65 M, density 1.18 g/L), and filter papers (pore size, 0.45 *μ*m) were supplied by Jimma University Laboratory of Drug Quality (JuLaDQ).

### 2.5. Test Methods

The quality control laboratory tests were performed in JuLaDQ. The laboratory tests were carried out according to the general and individual monographs (for tablet and capsule samples) specified in United States Pharmacopoeia. Instrument performance and system suitability tests were successfully performed for the analytical instruments and HPLC methods, respectively. Quality testing parameters based on which the products were evaluated as described in conventional monographs were (i) physical tests, uniformity of weight, friability, and tablet hardness, (ii) chemical tests for content of active ingredients and identity, and (iii) in vitro dissolution tests.

### 2.6. Physical Tests

As a comparison with the authentic drug product is always preferred, the first step in evaluating the quality of medicine is checking the packaging/labeling and dosage form of the sampled medicine. It aids in identifying suspicious products. Before we have begun lab tests, visual inspection was performed on both dosages form samples. Tablets must be able to withstand the rigors of handling and transportation experienced in the manufacturing plant, the drug distribution system, and the field at the hands of the end-users (patients/consumers). Doxycycline hyclate tablets were tested for hardness as per its monograph. Tablets are subjected to tumbling which is consistent with the level and time encountered during manufacture, dedusting, coating, handling, transport, and packaging and of course with the patient. The test measures the way tablets hold up under tumbling conditions. The stress of tumbling and resistance of chipping and abrasion is measured by the weight loss of the tablets, after testing, and the parameter applied to test this phenomenon is friability. Doxycycline hyclate tablets as well are challenged for the friability test according to the general pharmacopoeial specification. Uniformity of the dosage unit is the degree of uniformity in the amount of the drug substance among dosage units. It can be demonstrated through either content uniformity (by assay test) or weight variation. Doxycycline hyclate capsules and tablets were evaluated for weight variation.

### 2.7. Chemical Tests

The assay test is a critical quality attribute required to confirm that the labeled amount of drug is available in the given dosage form. The study samples were assayed for their content of doxycycline hyclate according to their individual monographs described in the USP 38. The samples were tested for identity via chromatographic peak retention times comparison of RS and test samples.

### 2.8. In Vitro Dissolution Tests

The dissolution test is intended to measure the time required for a given drug in an oral solid dosage form to go into solution under a specified set of conditions. It is a key analytical test used for the following: (a) formulation and optimization decisions: during product development, for products where dissolution performance is a critical quality attribute, both the product formulation and the manufacturing process are optimized based on achieving specific dissolution targets; (b) equivalence decisions: during generic product development and also when implementing a postapproval process or formulation changes, the similarity of in vitro dissolution profiles between the reference product and its generic or modified version is one of the key requirements for regulatory approval decisions; (c) product compliance and release decisions: during routine manufacturing, dissolution outcomes are very often one of the criteria used to make product release decisions [22, 23]. These and other vital roles make the test an in vitro fundamental analytical quality control method.

### 2.9. Dissolution Profile Comparison

The dissolution profile is a graphical representation (in terms of concentration against time) of the complete release of API from a dosage form in an appropriately selected dissolution medium. It reflects the API release pattern under the selected condition sets. Its evaluation of solid dosage forms provides a better characterization of the dissolution properties of that product [24].

Dissolution profile comparison helps to assure the similarity in the product performance and green signals to bioequivalence. Most importantly, it can allow making the appropriate necessary change in test formulation to achieve the same profile of the comparator product/brand leader provided that the dissolution profile of a particular product of the comparator product/brand leader is known [25]. So, to compare the dissolution profiles test samples and also attempt to identify the nature of API release, we have employed some approaches like ANOVA approach, model-independent approach (difference factor (*f*_1_) and similarity factor (*f*_2_)), and model-dependent approach (Weibull release model).

### 2.10. Statistical Analysis

The dissolution data were subjected to one-way ANOVA statistical analysis at 95 % CI followed by a post hoc test and two-tailed *t*-test. The dissolution data for 100 mg doxycycline capsule and tablet brands were compared to their respective innovator brands and differences were considered significant when *P* < 0.5.

### 2.11. Model-Independent Approaches

Model-independent approaches promote a direct comparison of the dissolution data, and the objective is essential to translate either the profile or profile differences into a single value. Consequently, the results do not depend on the selection of the specific parameter for fitting data but on the chosen sampling time *t*_*i*_ (*i* *=* 1,…, *n*) in the calculation. Model-independent approaches include ratio tests, dissolution efficiency, and fit factors.

(i) Fit factors
Fit factor uses a difference factor (*f*_1_) and a similarity factor (*f*_2_) was proposed to compare dissolution profiles [25]. The difference factor (*f*_1_) calculates the percent difference between the two curves at each time point and is a measure of the relative error between the two curves.
  (1) $$\begin{array}{c} f_{1}=\left\{\frac{\sum_{i=1}^{n}\left|R_{t}-T_{t}\right|}{\sum_{i=1}^{n}R_{t}}\right\}\times 100, \end{array}$$
where *n* is the number of time points, *R*_*t*_ is the dissolution value of the reference formulation at time *t*, and *T*_*t*_ is the dissolution value of the test formulation at time *t.*
The similarity factor (*f*_2_) is a logarithmic reciprocal square root transformation of the sum of squared error and is a measurement of the similarity in the percent (%) dissolution between the curves.
  (2) $$\begin{array}{c} f_{2}=50\;\log\left\{\frac{1}{\sqrt{1+\left(1/n\right)\sum {\left(R_{t}-T_{t}\right)}^{2}}}\right\}\times \;100. \end{array}$$
The values of *f*_1_ range from 0 to 15 while *f*_2_ ranges from 50 to 100. A test product is similar and hence equivalent to a reference product if *f*_1_ ≤ 15 and *f*_2_ ≥ 50. Also, two products are dissimilar and hence nonequivalent when *f*_1_ > 15 and *f*_2_ < 50 [25, 26].
(ii) Dissolution efficiency (DE)
It is defined as the area under the dissolution curve up to a certain time (*t*), expressed as a percentage of the area of the rectangle described by 100% dissolution at the same time point [27–29]. It is obtained using the following equation:
  (3) $$\begin{array}{c} \text{DE}=\frac{\int_{t1}^{t2}y\text{ d}t\;}{y100\left(t2-t1\right)}\times 100, \end{array}$$
where *y* is the percentage of the dissolved product.
The integral of the numerator which is the area under the curve was calculated using the trapezoidal method [30].
  (4) $$\begin{array}{c} \text{AUC}=\;\sum_{i=1}^{n}\frac{\left(t_{1}-t_{i-1}\right)\left(y_{i-1}-y_{i}\right)}{2}\;, \end{array}$$
where *t*_*i*_ is the *i*th time point and *y*_*i*_ is the % of dissolved product at time *t*_*i*_.
(iii) Ratio test approach
Ratio tests are performed as ratios of percent drug dissolved, DE, and mean dissolution times of the reference formulation with those of a test formulation at the same sampling time [27]. The most common ratio test is performed by comparison of two mean dissolution times (MDTs), which are calculated by
  (5) $$\begin{array}{c} \text{MDT}=\frac{\sum_{i}^{n}{\bar{t}}_{i}\Delta M_{i}}{\sum_{i}^{n}\Delta M_{i}}, \end{array}$$
where *i* is the sample number, *n* is the number of dissolution sample times, $\bar{t}=\left(t_{i}-1+t_{i}\right)/2$ is the time at the midpoint between *t*_*i*−1_ and *t*_*i*_, and Δ*M*_*i*_ is the additional amount of drug dissolved between *t*_*i*−1_ and *t*_*i*_. More precisely, the equation will be rearranged to
  (6) $$\begin{array}{c} \text{MDT}=\;\frac{\sum t_{I}\Delta M_{i}}{M_{\infty }}, \end{array}$$
where *t*_*i*_ is an intermediate time of the intervals of sampling time, Δ*M*_*i*_ is the amount of API dissolved in every interval of *t*, and M_∞_ is the maximum of API dissolved.
The DE and the MDT of each of the products were calculated using KineticDS3 software. The dissolution profiles of the products along with their respective sampling points were fed individually to the software.

### 2.12. Model-Dependent Methods

Model-dependent methods explore the mathematical equations governing the liberation profile as a function of certain parameters related to the pharmaceutical dosage form. These models allow an easy quantitative interpretation of data. Different mathematical models have been proposed to analyze the dissolution profiles through which the mechanism of drug release can be determined. The mathematical models of a dissolution profile can be deduced by a theoretical analysis of the process, such as zero-order kinetics, first-order kinetics, Hixson–Crowell, and Weibull models. In this work, doxycycline drug release kinetics was analyzed by them considering the amounts of drug released up to 90 min [31–33].

(i) Zero-order model
Dissolution of the drug from pharmaceutical dosage forms that do not disaggregate and release the drug slowly can be represented by the following equation:
  (7) $$\begin{array}{c} Q_{t}=\;Q_{0}-k_{0}t, \end{array}$$
where *Q*_0_ is the initial amount of drug in the pharmaceutical dosage form, *Q*_*t*_ is the amount of drug in the pharmaceutical dosage form at time *t*, and *K* is proportionality constant.
The pharmaceutical dosage forms following these profiles release the same amount of drug by the unit of time and it is the ideal method of drug release in order to achieve a pharmacological prolonged action.
(ii) First-order model
The application of this model to drug dissolution studies was first proposed by Perrier [34]. The release of the drug which followed first-order kinetics can be expressed by the following equation:
  (8) $$\begin{array}{c} \log\;Q_{t}=\log\;Q_{0}-\frac{kt}{2.303}, \end{array}$$
where *Q*_0_ is the initial concentration of the drug, *k* is the first-order rate constant, and *t* is the time. The data obtained are plotted as log cumulative percentage of drug remaining versus time which would yield a straight line with a slope of −*k*/2.303.
(iii) Hixson–Crowell model
Drug powder has uniformed size particles; Hixson and Crowell derived the equation which expresses the rate of dissolution based on the cube root of the weight of particles and the radius of a particle is not assumed to be constant.
This is expressed by the following equation:
  (9) $$\begin{array}{c} M_{0}^{1/3}-\;M_{t}^{1/3}=кt, \end{array}$$
where *M*_0_ is the initial amount of drug in the pharmaceutical dosage form, *M*_*t*_ is the remaining amount of drug in the pharmaceutical dosage form at the time “*t*,” and *к* is proportionality constant incorporating the surface-volume relation. The equation describes the release from systems where there is a change in surface area and diameter of particles or tablets [34]. To study the release kinetics, data obtained from *in vitro* drug release studies were plotted as the cube root of drug percentage remaining in matrix *versus* time.
(iv) Weibull model
This model has been described for different dissolution processes as the following equation:
  (10) $$\begin{array}{c} M=M_{0}\left\{1-e^{{\left(t-T\right)}^{\beta }/\alpha }\right\}, \end{array}$$
where *M* is the amount of drug dissolved as a function of time *t. M*_0_ is the total amount of drug being released. *T* accounts for the lag time measured as a result of the dissolution process. Parameter *α* denotes a scale parameter that describes the time dependence, while *β* describes the shape of the dissolution curve progression.
The model is more useful for comparing the release profiles of matrix type drug delivery.

## 3. Results and Discussion

A total of ten doxycycline capsules and tablets samples were collected between July and June 2016 from Jimma Town licensed pharmaceutical retail outlets (Table 1). Of these, 3 samples were tablets and 7 were capsules. The products were manufactured locally and imported from Asia and Europe.

The results of the different quality control tests of the samples are presented in Table 2 and are detailed below.

*Visual Inspection*. Neither the dosage forms nor the dosage units of any of the samples inspected were found defective. No defect in packaging and labeling was encountered.

*Identification*. All samples had the intended active ingredient as demonstrated by comparing the retention time of the standard and the samples.

*Assay*. The assay values for doxycycline hyclate capsules ranged from 93.40 to 116.00% lc (mean: 99.3%), while that of tablets ranged from 92.60 to 119.62% lc (mean: 104.74%). According to USP 38, doxycycline hyclate capsules and tablets contain not less than 90.0 percent and not more than 120.0 percent of the labeled amount of doxycycline API. Although the study samples comply with the standard for assay, 4 products have assay values more than 5% below the label claim (lc) and 2 products have assay values more than 15% of the target values of the manufacturers.

To identify whether there is a significant variation in the assay values of the tested products, the assay values of the products was checked by single-factor ANOVA. The test indicated that there is a significant variation between the amounts of API contents of the different brands.

*Dosage Uniformity*. Dosage uniformity is measured to ensure a constant dose of drugs between individual dosage forms. All doxycycline hyclate capsules (Table 2) and tablet (Table 3) samples were in line with pharmacopoeial acceptance criteria for uniformity of weight test. The average weight of net weight of capsules ranges from 127.22 mg to 362.68 mg. The expected strength of doxycycline is 100 mg. The variation in average net weights of the capsules studied reveals that different manufacturers use different and/or the same kind(s) of excipients in different proportions in their products. The comparator product (D03) has about 183.91 mg of various excipients in it.

The standard deviation which is a measure of variability or dispersion around the mean weight of the twenty capsules sampled was lowest for D05 (±1.74) and highest for D07 (±9.25) whereas the mean weight of the twenty tablets sampled was lowest for D09 (±1.54) and highest for D10 (±2.13). Thus, tablets (D09) had the best uniformity of weight variation while capsules (brand D07) had the highest dispersion/least clustering of samples weight around the mean weight and hence the least uniform brand. The highest dispersion from the mean for the tablet brand D10 was almost equal to the lowest variation from the mean for the capsule brand D01. However, the percent deviation of all samples was less than the upper acceptance limits and 90% of the tested products were more persistently uniform than the specifications of the European Pharmacopoeia.

Variability in tablet weight could be the result of defective formulation and production processes such as poor weighing of active pharmaceutical ingredients and excipients, poor mixing of ingredients, and changes in tablet compression force applied, while in capsule weight, variations arise due to formulation practices that do not comply with good manufacturing practices—the vibrations of a capsule filling machine, capsule filling speed, flowability, particle size, the compressibility, air permeability, powder density, and environmental conditions—and lead to variation in the content of capsule [35].

A variation in tablet/capsule weight could be an indication of a change in the content of API in the drug products. Such formulations expose the patients to pharmacodynamic and pharmacokinetic fluctuations, on consumption.

*Friability Test*. This test was done for doxycycline hyclate tablet samples (Table 2). One product brand D_**09**_ was found to be highly friable that cannot hold up under tumbling conditions and failed to meet the pharmacopeial specification for the friability test. Its percent weight loss was 13.78%.

*Hardness Test*. Doxycycline hyclate tablet samples were challenged for this test (Table 2). A product that has failed the friability test again was unable to withstand the minimum crushing force applied. However, it has been provided adequate protection by presenting a single strip of 10 tablets (sandwiched in a folded leaflet) in a hard cartoon in a way that withstands the rigors of handling and transportation experienced in the drug distribution and the field at the hands of the end-users.

It is known that the compression force during a tableting process plays an essential role in the overall properties of the products, such as tablet disintegration rate, friability, and hardness. It is therefore concluded that the compression forces used during the manufacturing processes for brands D08 and D10 are likely to be significantly higher than those used for brands D09.

*Dissolution*. Dissolution is the process of extracting the API out of the dosage from the solid-state matrix into a solution within the gastrointestinal tract. The dissolution of doxycycline hyclate 100 mg capsule brands was rapid with the release of more than 85% of the labeled amount within 30 min. All capsule samples passed the single-point dissolution test specification. Guidelines discourage further dissolution profile evaluation. However, we tried to see dissolution profile evaluation in different methods.

Unfortunately, the dissolution test for the tablets was not within the regulatory limits for the definition of immediate-release (IR) specifications. Only after 75 minutes, more than 85% of the label claimed the content of the drug released.

**Figure 1:**
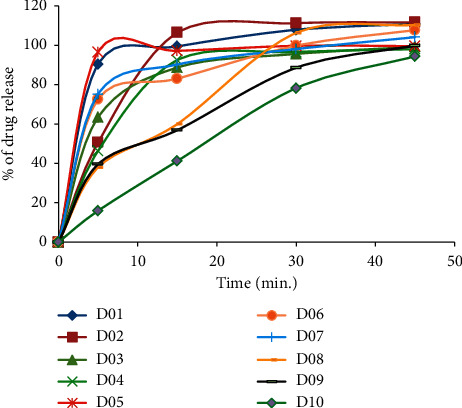
Drug release profiles of 100 mg strength doxycycline hyclate in phosphate buffer ph 6.8 (mean ± SD, *n* = 3).

As shown in Figure 1, drug release at specified points was highly variable. They exhibited different drug release patterns at different time points. For products D03, D04, D06, and D07, within the first 30 minutes, the fraction they released reached ≥95% and fulfilled the regulatory limits for the definition of rapid dissolution. The remaining capsule samples D01, D02, and D05 released ≥95% of their API before the first 15 minutes has been elapsed and showed to possess very rapid dissolution.

### 3.1. Dissolution Profile Comparison

To check whether there is a difference in the release profile of brands, one-way ANOVA, single-factor statistical analysis at 95% CI was used. The SPSS result showed that there was no significant difference between the released amounts of drugs from different brands. To confirm the absence of significant variation, one-way ANOVA post hoc test and two-tailed *t*-test for two independent samples were applied. The result showed that the test products and comparators have no significant difference in their release profiles.

Additionally, in order to demonstrate the equivalence of all doxycycline capsules and tablets brands with comparator products, other approaches were employed.

Fit factor (*f*_1_ and *f*_2_ factors) was performed for six capsules and two tablets brands using their respective comparators D03 and D08 as reference (Table 2). The calculated results for the D10 tablet brand *f*_2_ value were <50 and *f*_1_ was >15. Brand D09 had an *f*_2_ value <50 and *f*_1_ <15, which is outside the acceptable range specified by the USFDA [26]. The tablet brands were dissimilar to the comparator and with one another, because *f*_2_ value was <50 for all the products. Therefore, the tablets are not interchangeable, while capsules D04, D06, and D07 had *f*_2_ value >50 and *f*_1_ <15 and for D01 and D05, the *f*_2_ value was <50 and *f*_1_ was <15. As per fit factor specifications, only brands D04, D06, and D07 were interchangeable and equivalent with D03. The remaining capsule brands were unable to fit this category since their *f*_2_ value was <50.

Furthermore, interchangeability and/or equivalence was confirmed by comparing DE. DE up to 90 minutes was calculated from the dissolution profile of all brands of doxycycline. As listed in Table 3, except D04, all tested capsule doxycycline brands had high DE than the comparator. To decide which product is equivalent to the comparator, their DE difference has to be within ±10 [36]. Accordingly, all products were equivalent to D03 as the difference percent (test product-comparator product) is <10. Tested tablet samples had generally low DE compared to capsules. For brand D10, DE difference exceeded the acceptance limit (difference of 12.2%). It cannot be considered interchangeable with the D08.

In quality control studies, passing for other quality control tests, it has been revealed that some drugs fail equivalence/interchangeability test [37]. This may be largely related to polymorphism and choice of the crystalline form for drug making as they cause differences in physical properties as solubility and dissolution rates [38]. But no polymorphism of doxycycline hyclate has been reported [11]. The unbalanced amphoteric character of D10 may expose its failure [39].

The mean dissolution time was another test category within the model-independent method that was employed in this paper. It is determined from the accumulative curves of dissolved API as a function of time. The MDT values of D03, D04, D06, and D07 and D01, D02, and D05 had similar dissolution profiles before 30 min. Their MDT values greatly varied after 30 minutes. Brands D01 and D05 showed significant deficiencies compared with those of brands D02, D03, D04, D06, and D07. The MDT values of tablets throughout the run were variable. On the other hand, except for D04, all tested brands had an MDT ratio greater than one. This indicates that those brands have a higher rate of dissolution than the comparators.

The dissolution profiles corresponding to the comparators and other products were evaluated by fitting experimental data to the model-dependent models, the zero-order, the first-order, the Hixson–Crowell, and the Weibull models. The model that gives a high correlation coefficient, *r*^*2*^ value, is considered as the best fit of the release data [40]. In Table 4, among the five models fitted to each dissolution profile, the Weibull model provided the best adjustment curve for all brands, with the higher correlation coefficients. The best fittings were obtained with brands D01, D05, and D10, with maximum determination coefficients. Beyond this, all investigated brands followed the same release mechanism.

## 4. Conclusions

Doxycycline is a biowaiver product. Hence *in vitro* dissolution evaluation suffices its market approval. This work, however, found equivalence problems between “comparators” and interchangeability issues among doxycycline different brands already distributed in the market. It is an alarm to look at the dossier evaluations and legal approval mechanism.

In this quality assessment study, however, the samples passed quality control tests, except D09 brand which failed friability; it has been revealed that five (D01, D02, D05, D09, and D10) of eight brands had problems with interchangeability. Only three (D04, D06, and D07) doxycycline hyclate brands were found to be equivalent to the comparators. Doxycycline hyclate (D03 and D08) was comparators used for equivalence/interchangeability study of capsules and tablets, respectively.

## Figures and Tables

**Table 1:** Profile of doxycycline hyclate 100 mg capsule and tablet brands marketed in Jimma, Ethiopia.

Sample code	Brands name	Supplier	Batch number	Mfg date	Expiry date	Origin
D01	Teradoxin	Huons Co., Ltd.	CBC 503	09/2015	09/2018	S. Korea
D02	Medomycin	Medochemieltd., Limassol-Cyprus Europe	APL028	11/2013	11/2017	Cyprus
D03	Doxylagap	Labatec-Pharma SA Meyrin for lagap SA Vezia/Switzerand	2708	11/2014	11/2017	Switzerand
D04	Doxyleb	Leben LaboratoriesPVt. LTD	C-106	04/2016	03/2018	India
D05	Epadoxine	East African pharmaceuticals	F1202	*∗*	11/2018	Ethiopia
D06	Doxycap	Addis pharmaceuticals Factory	20644	*∗*	07/2020	Ethiopia
D07	Doxycad	Cadila pharmaceuticals Ltd	014013BX58	05/2016	04/2018	Ethiopia
D08	Doxy denk	Artesan Pharma GmbH & Co. KG Prinzregentenstr. Germany	2957	09/2015	08/2018	Germany
D09	Miraclin	Laboratorio Farmacologico Milanese S.r.l.-Italy	010216	02/2016	02/2019	Italy
D10	Remycin	Remedica, Limassol-Cyprus Europe	68832	06/2016	06/2021	Cyprus

D01–D07 are capsule dosage forms. D08–D10 are tablet dosage forms. *∗*Not found. Mfg: manufacturing.

**Table 2:** Pharmacopoeial quality test results for doxycycline hyclate 100 mg brands.

Sample code	Assay (±SD), *n* = 20	Compliance with USP assay test	Average weight (gm), *n* = 10 for caps and 20 for tabs (±SD)	Compliance with Int. Ph for caps, USP for tabs weight variation test	Friability (%), *n* = 10	Compliance with USP friability test	Average hardness in Newton	Compliance with USP hardness test
D01	97.70 (0.06)	Passed	362.68 (2.68)	Passed				
D02	116.0 (0.16)	Passed	330.86 (3.98)	Passed				
D03	99.40 (0.13)	Passed	283.91 (3.63)	Passed				
D04	94.70 (0.13)	Passed	221.08 (3.36)	Passed				
D05	93.40 (0.23)	Passed	127.22 (1.74)	Passed				
D06	94.60 (0.05)	Passed	222.54 (7.03)	Passed				
D07	99.30 (0.09)	Passed	140.32 (9.25)	Passed				
D08	119.6 (0.07)	Passed	259.07 (1.85)	Passed	0.17	Passed	54.00 (4.06)	Passed
D09	102.0 (0.21)	Passed	264.97 (1.68)	Passed	13.78	Failed	31.83 (2.07)	Failed
D10	92.60 (1.82)	Passed	211.98 (2.13)	Passed	0.01	Passed	83.64 (6.49)	Passed

Caps: capsules; tabs: tablets; USP: United State Pharmacopeia; Int. Ph: International Pharmacopeia.

**Table 3:** Model-independent approaches results of doxycycline hyclate 100 mg brands.

Sample code	*f*_2_	*f*_1_	DE (%)	%DE difference	MDT	MDT ratio	Mean % release ratio	SD % release ratio	RSD % release ratio
D01	64	13	93.3	2.9	3.01	0.66	1.13	0.07	5.71
D02	40	18	90.3	0.1	4.32	0.88	1.08	0.19	17.47
D04	57	7	86.3	4.1	6.19	1.04	0.94	0.14	15.27
D05	38	14	94.1	3.7	2.68	0.45	1.17	0.24	20.52
D06	57	8	84.0	6.4	7.20	0.95	1.05	0.09	8.10
D07	59	6	87.1	3.3	5.81	0.83	1.07	0.08	7.39
D09	41	10	69.9	2.3	3.61	1.09	0.91	0.06	1.09
D10	29	22	61.0	11.2	4.18	1.23	0.77	0.04	1.23

SD: standard deviation; RSD: relative standard deviation. %DE—D03, 90.4%; D08, 72.2. MDT—D03, 4.22; D08, 3.33.

**Table 4:** Model-dependent approaches results of doxycycline hyclate 100 mg brands when correlation coefficients were considered.

Models	Sample code									
	D01	D02	D03	D04	D05	D06	D07	D08	D09	D10
Zero order	0.3208	0.4282	0.4282	0.5589	0.2899	0.5939	0.5165	0.8702	0.8962	0.9428
First order	0.2746	0.2808	0.2790	0.2893	0.2734	0.2844	0.2817	0.8044	0.2271	0.7001
Hixson–Crowell	0.2894	0.3402	0.3294	0.4088	0.2785	0.3829	0.3559	0.6789	0.6843	0.7281
Weibull	0.9980	0.9946	0.9958	0.9926	0.9985	0.9924	0.9939	0.9807	0.9873	0.9986

## Data Availability

All essential data are included in the manuscript.
